# Risk analysis of intracranial aneurysm rupture based on the arterial segment of origin

**DOI:** 10.3389/fneur.2024.1339144

**Published:** 2024-08-21

**Authors:** Junqiang Feng, Yudi Tang, Wei You, Yuhua Jiang, Zhengkun Xu, Yan Zhao, Xinke Liu, Jian Lv, Peng Liu, Haining Wei, Mahmud Mossa-Basha, Youxiang Li, Yang Wang, Chengcheng Zhu

**Affiliations:** ^1^Department of Neurosurgery, Beijing Chaoyang Hospital, Capital Medical University, Beijing, China; ^2^Department of Interventional Neuroradiology, Beijing Neurosurgical Institute and Beijing Tiantan Hospital, Capital Medical University, Beijing, China; ^3^School of Mechanical Engineering, Hebei University of Technology, Tianjin, China; ^4^School of Mechanical Engineering, University of Science and Technology Beijing, Beijing, China; ^5^Center for Biomedical Imaging Research, Department of Biomedical Engineering, Medical School, Tsinghua University, Beijing, China; ^6^Department of Radiology, University of Washington, Seattle, WA, United States

**Keywords:** aneurysm, aneurysm rupture risk, aneurysm rupture, risk assessment, CT angiography

## Abstract

**Background and objective:**

The rupture risk of intracranial aneurysms (IAs) is related to their arterial origin, but whether the different segments of the artery have different risks and act as independent risk factors is still unknown. Our study aimed to investigate the rupture risk of IAs in different arterial segments in a large Chinese cohort.

**Methods:**

Imaging and clinical data of consecutive patients with IAs diagnosed by Computed Tomography angiography (CTA) from January 2013 to December 2022 were collected. Two neuroradiologists independently identified ruptured and unruptured IAs based on imaging and medical records. The internal carotid artery (ICA), middle cerebral artery (MCA), anterior cerebral artery (ACA), vertebral artery (VA), and posterior cerebral artery (PCA) were segmented according to the Bouthillier and Fischer segmentation methods. Stenoses of the proximal parent vessel were evaluated and documented. The Institutional Review Board (IRB) at Beijing Tiantan Hospital approved this retrospective study.

**Results:**

A total of 3,837 aneurysms {median size 3.5 mm [interquartile range (IQR) 2.6–5.1 mm]; 532 ruptured} were included in this study from 2,968 patients [mean age: 57 years (IQR 50–64); male patients: 1,153]. Ruptured aneurysms were most commonly located in the posterior inferior cerebellar artery (PICA) (52.9%), anterior communicating artery (ACoA) (33.8%), other locations (33.3%), ACA (22.4%), and basilar artery (BA) (21.4%). The locations with the highest likelihood of rupture were the C7 ICA (21.3%), M2 MCA (24.0%), distal MCA (25.0%), and A2 ACA (28.1%). IAs originating from the C7 (*p* < 0.001), dM1 (*p* = 0.022), and dA1 (*p* = 0.021) segments were independent risk factors for rupture. IAs without stenosis of the proximal parent vessel were associated with a higher risk of rupture (*p* = 0.023).

**Conclusion:**

There are unique associations between the origins of aneurysms from various arterial segments. Aneurysms originating from the anterior communicating artery (ACoA), BA, PICA, A2, dA, C7, and M2 indicate a higher risk of rupture. Aneurysms originating from C4, C5, and C6 indicate a lower risk of rupture. C7 IAs, ACoA IAs, and PICA IAs seem to be independent risk factors.

## Introduction

Intracranial aneurysms (IAs) are a common vascular pathology found in roughly 2–3% of the general population (1, 2). In most cases, IAs are asymptomatic and the rupture rate is very low (≤ 1% per year) (3). IA rupture can, however, lead to subarachnoid hemorrhage (SAH), which is associated with high mortality and morbidity (case fatality ≈ 40%) (3). Although many factors have shown associations with IA rupture in cohort studies (4–6), their predictive ability remains limited with moderate accuracy (7). A more accurate assessment of IA rupture risk factors is needed.

The origin of an aneurysm has been associated with aneurysm rupture (5). Aneurysms originating from the anterior communicating artery (ACoA) and posterior circulating artery (PCA) are usually considered to have a high risk of rupture (8). IAs originating from different segments of the same artery may have different rupture risks (9), but it has been rarely studied. Our study aimed to investigate the rupture risk of IAs in different artery segments in a large Chinese cohort.

## Materials and methods

### Subjects

The Institutional Review Board (IRB) at Beijing Tiantan Hospital approved this retrospective study with a waiver of informed consent. Imaging data and clinical baseline data of 2,968 patients with saccular IAs detected on head CTA at Beijing Tiantan Hospital from January 2013 to December 2022 were included in this study. Patients with incomplete medical records, cerebral vascular malformations, moyamoya disease, or traumatic or inflammatory IAs were excluded. In addition, patients with a history of subarachnoid hemorrhage were not included because of incomplete medical data. Patients' demographics including age, gender, blood lipids, blood glucose, blood pressure, and smoking history were recorded.

### Grouping

All data were anonymized before processing. Two neuroradiologists with 12 and 13 years of experience independently identified ruptured and unruptured aneurysms based on existing CTA images according to previously developed criteria, which included the identification of the rupture site in patients with SAH and multiple aneurysms (10). In case of disagreement on rupture status, a third neuroradiologist with 21 years of experience adjudicated according to the imaging, medical record, and his experience. Finally, all data were divided into a ruptured and an unruptured group. The proportion of the ruptured aneurysms was defined as the ratio of ruptured aneurysms on a certain artery (or a certain segment) to all aneurysms on this artery (or this segment).

### Location and image feature acquisition

#### Location standard

Arteries that originate aneurysm were documented, which included the internal carotid artery (ICA), anterior cerebral artery (ACA), anterior communicating artery (ACoA), anterior choroidal artery (AchoA), ophthalmic artery (OA), middle cerebral artery (MCA), posterior cerebral artery (PCA), posterior communicating artery (PcoA), basilar artery (BA), vertebral artery (VA), posterior inferior cerebellar artery (PICA), superior cerebellar artery (SCA), and anterior inferior cerebellar artery (AICA).

The ICA was divided into seven segments according to the Bouthillier segmentation method (11) (cervical segment C1, petrous segment C2, lacerum segment C3, cavernous segment C4, clinoid segment C5, ophthalmic segment C6, and communicating segment C7). The MCA was divided into five segments according to the Fischer segmentation method (horizontal segment M1, insular segment M2, opercula segment M3, and cortical segments M4 and M5). The ACA was divided into five segments according to the Fischer segmentation method (12) (suprachiasmatic segment A1, subcallosal segment A2, genu segment A3, the frontal lobe segment of the pericallosal artery A4, and the parietal lobe segment of the pericallosal artery A5). The VA was divided into four segments (pre-foraminal segment V1, foraminal segment V2, external spinal segment V3, and intradural segment V4).

The aneurysms were less distributed in some locations; thus, C1–C3 were merged into pC, M3–M5 were merged into dM, A3–A5 were merged into dA, and the SCA, AICA, and OA were merged into others. Considering that there were many aneurysms at the bifurcation of C7 and PcoA, we merged them into PcoA-C7 for further analysis.

In the analysis of the independent risk factors of the MCA, ACA, and PCA, M2–M5 were merged into dM1, A2–A5 were merged into dA1, and P2–M4 were merged into dP1.

#### Feature acquisition

The cerebral arteries were reconstructed from bone-subtracted CTA images using a 3D slicer (Version 4.10.1). The two neuroradiologists independently determined the origin of the aneurysms, whether it was a bifurcation aneurysm and whether it had a daughter sac, as well as the degree of stenosis of the proximal parent artery (no, mild <50%, moderate 50–70%, severe 70%, occluded) using reconstructed three-dimensional CTA datasets. In case of any interrater discrepancies, the senior neuroradiologist adjudicated. The aneurysm size was measured as the maximum height from the midpoint of the neck to the dome of the aneurysm.

### Statistical analysis

Normally distributed data and non-normally distributed data were expressed as mean ± standard deviation and median (interquartile range), respectively. Categorical data were expressed as percentiles. The proportion of each factor was statistically compared between the ruptured group and the unruptured group to evaluate its correlation with rupture. A *t*-test was used for the parameters of the normally distributed data, and a Mann–Whitney *U*-test was used for the parameters of the non-normally distributed data. Pearson's chi-square test, logistic regression analysis, and propensity score matching (PSM)(Match Tolerance = 0.001) were used to assess the correlation between the risk factors and the ruptured aneurysms. Pearson's chi-square test with continuity correction and the Fisher's exact test were used to assess the correlation between the aneurysm location and rupture risk. *t*-tests were also used to assess the correlation between the aneurysm origin and risk factors. PSM was performed between the ruptured and unruptured groups with a 1:1 ratio. Differences with a *p*-value of <0.05 were considered significant. Data were analyzed with SPSS 23.0 software (SPSS Inc, Chicago, IL).

## Results

A total of 3,837 aneurysms (ruptured 532) from 2,968 patients [age 57 (50–64)] were included. More detailed characteristics are shown in Tables 1, 2. The aneurysm size and location are shown in Table 3.

**Table 1:** Characteristics of IAs patients.

	**Ruptured (*n* = 521)**	**Unruptured (*n* = 2,156)**	***p*-value**
**Sex**			
Male	42.23%	38.22%	0.092
Female	57.77%	61.78%	
**Number of aneurysms**			
Single	79.08%	77.88%	0.551
Multiple	20.92%	22.12%	
Age	57 (50, 65)	57 (50, 63)	0.214
**Cerebral artery**			
Non-stenotic	35.70%	44.71%	0.004
<50%	37.24%	31.22%	
50–75%	11.90%	9.74%	
75–100%	6.91%	7.33%	
Occlusion	8.25%	7.00%	
**Blood lipids**			
Hyperlipidemia	8.64%	20.18%	<0.001
Non-hyperlipidemia	91.36%	79.82%	
**Blood glucose**			
Diabetes	15.16%	15.82%	0.713
Non-diabetes	84.84%	84.18%	
**Blood pressure**			
Hypertension	64.30%	66.60%	0.318
Non-hypertension	35.70%	33.40%	
**Smoking history**			
Smoking	26.30%	24.44%	0.380
Non-smoking	73.70%	75.56%	

*p*, chi-squared test or Mann–Whitney *U*-test.

**Table 2:** Characteristics of the aneurysms and logistic regression results between the two IA groups.

	**Ruptured, % (*n* = 521)**	**Unruptured, % (*n* = 2,824)**	***p***	*p* ^*^	**OR (95%CI)**
**Sex**					
Male	42.23%	37.96%	0.066	-	-
Female^#^	57.77%	62.04%			
**Number of aneurysms**					
Single	77.74%	57.37%	<0.001	<0.001	2.828 (2.194–3.645)
Multiple^#^	22.26%	42.63%			
**Daughter aneurysms**					
Without^#^	69.48%	97.98%	<0.001	<0.001	20.119 (14.073–28.762)
With	30.52%	2.02%			
**Situation**					
Non-bifurcation^#^	40.69%	66.15%	<0.001	<0.001	2.267 (1.827–2.813)
Bifurcation	59.31%	33.85%			
Size	5.11 (3.85, 7.09)	3.385 (2.52, 4.80)	<0.001	0.001	1.049 (1.020–1.079)
Age	57 (50, 65)	58 (51, 64)	0.974	–	–
**Parent artery**					
Non-stenotic	87.52%	78.40%	<0.001	0.011	1.506 (1.099–2.063)
Stenotic^#^	12.48%	21.60%			
**Blood lipids**					
Hyperlipidemia	8.64%	19.65%	<0.001	<0.001	2.729 (1.915–3.890)
Non-hyperlipidemia^#^	91.36%	80.35%			
**Blood glucose**					
Diabetes	15.16%	16.08%	0.601	–	–
Non-diabetes	84.84%	83.92%			
**Blood pressure**					
Hypertension	64.11%	66.18%	0.359	–	–
Non-hypertension	35.88%	33.82%			
**Smoking history**					
Smoking	26.30%	24.22%	0.312	–	–
Non-smoking	73.70%	75.78%			

^#^Contrast indicator.

*p*, Chi-squared test or Mann–Whitney *U*-test.

p^*^, logistic regression.

OR, odds ratio.

**Table 3:** The distribution of aneurysms of the two groups, the ruptured IAs' proportions, and the size of aneurysms of each location.

**Location**	**RAs/all RAs, % (*n*)**	**UAs/all UAs, %(*n*)**	***p***	**Ruptured IAs' proportion (95% CI), %**	**Median size IQR, mm**
Total	100.00% (532)	100.00% (3,305)	–	13.86% (12.80–14.99%)	3.53 (2.58, 5.17)
ACoA	24.06% (128)	7.59% (251)	<0.001	33.77% (29.19–38.67%)	4.29 (3.10, 6.20)
BA	5.64% (30)	3.33% (110)	0.008	21.43% (15.44–28.94%)	3.91 (2.585, 6.30)
VA	2.07% (11)	2.81% (93)	0.325	10.58% (6.01–17.96%)	3.925 (2.7725, 5.4975)
PICA	1.69% (9)	0.24% (8)	<0.001	52.94% (30.96–73.83%)	4.55 (3.935, 6.145)
ACA	9.21% (49)	5.14% (170)	<0.001	22.37% (17.35–28.34%)	3.22 (2.33, 4.88)
A1	1.69% (9)	1.94% (64)	0.701	12.33% (6.62–21.80%)	3.16 (2.265, 4.695)
A2	5.08% (27)	2.09% (69)	<0.001	28.13% (20.11–37.83%)	3.20 (2.10, 4.845)
dA	2.44% (13)	1.12% (37)	0.012	26.00% (15.87–39.55%)	3.785 (2.805, 4.9525)
ICA^#^	36.65% (195)	60.54% (2,001)	<0.001	8.88% (7.76–10.14%)	3.25 (2.49, 4.66)
pC	0.19% (1)	0.48% (16)	0.547	5.88% (1.05–26.98%)	4.47 (3.27, 7.315)
C4	0.94% (5)	7.99% (264)	<0.001	1.86% (0.80–4.28%)	2.80 (2.27, 4.09)
C5	0.94% (5)	6.08% (201)	<0.001	2.43% (1.04–5.56%)	3.305 (2.5675, 4.27)
ICA^*^	34.59% (184)	45.99% (1,520)	<0.001	10.80% (9.41–12.36%)	3.30 (2.50, 4.72)
C6	3.95% (21)	27.72% (916)	<0.001	2.24% (1.47–3.40%)	3.19 (2.51, 4.50)
C7	30.64% (163)	18.28% (604)	<0.001	21.25% (18.50–24.28%)	3.5 (2.50, 5.22)
MCA	17.48% (93)	17.16% (567)	0.854	14.09% (11.64–16.95%)	4.105 (2.95, 6.14)
M1	12.78% (68)	14.77% (488)	0.778	12.23% (9.76–15.22%)	4.07 (2.9525, 6.135)
M2	4.32% (23)	2.21% (73)	0.004	23.96% (16.53–33.39%)	4.635 (2.935, 6.2975)
dM	0.38% (2)	0.18% (6)	0.689	25.00% (7.15–59.07%)	4.18 (2.2875, 6.975)
PCA	2.26% (12)	2.12% (70)	0.839	14.63% (8.57–23.85%)	3.20 (2.67, 4.9325)
P1	1.13% (6)	1.12% (37)	0.987	13.95% (6.55–27.26%)	2.84 (1.81, 3.53)
P2	0.94% (5)	0.91% (30)	0.942	14.29% (6.26–29.38%)	4.03 (2.80, 7.17)
dP	0.19% (1)	0.09% (3)	1.000	25.00% (4.56–69.94%)	2.825 (1.695, 6.82)
PcoA	0.19% (1)	0.82% (27)	0.114	3.57% (0.63–17.71%)	3.905 (2.8675, 5.60)
PcoA-C7	30.83% (164)	19.09% (631)	<0.001	20.63% (17.96–23.58%)	3.52 (2.50, 5.22)
Other	0.75% (4)	0.24% (8)	0.124	33.33% (13.81–60.93%)	3.955 (2.8575, 5.1375)

RAs, ruptured intracranial aneurysms; UAs, unruptured intracranial aneurysms; ICA, internal carotid artery; ACA, anterior cerebral artery; ACoA, anterior communicating artery; MCA, middle cerebral artery; PCA, posterior cerebral artery; PcoA, posterior communicating artery; BA, basilar artery; VA, vertebral artery; PICA, posterior inferior cerebellar artery. pC, C1 to C3; dM, M3 to M5; dA, A3 to A5; other, superior cerebellar artery, anterior inferior cerebellar artery, and ophthalmic artery.

IQR, interquartile range.

^#^All aneurysms in the ICA.

^*^All intracranial aneurysms in the ICA.

### Distribution of IAs and EAs

**Figure 1:**
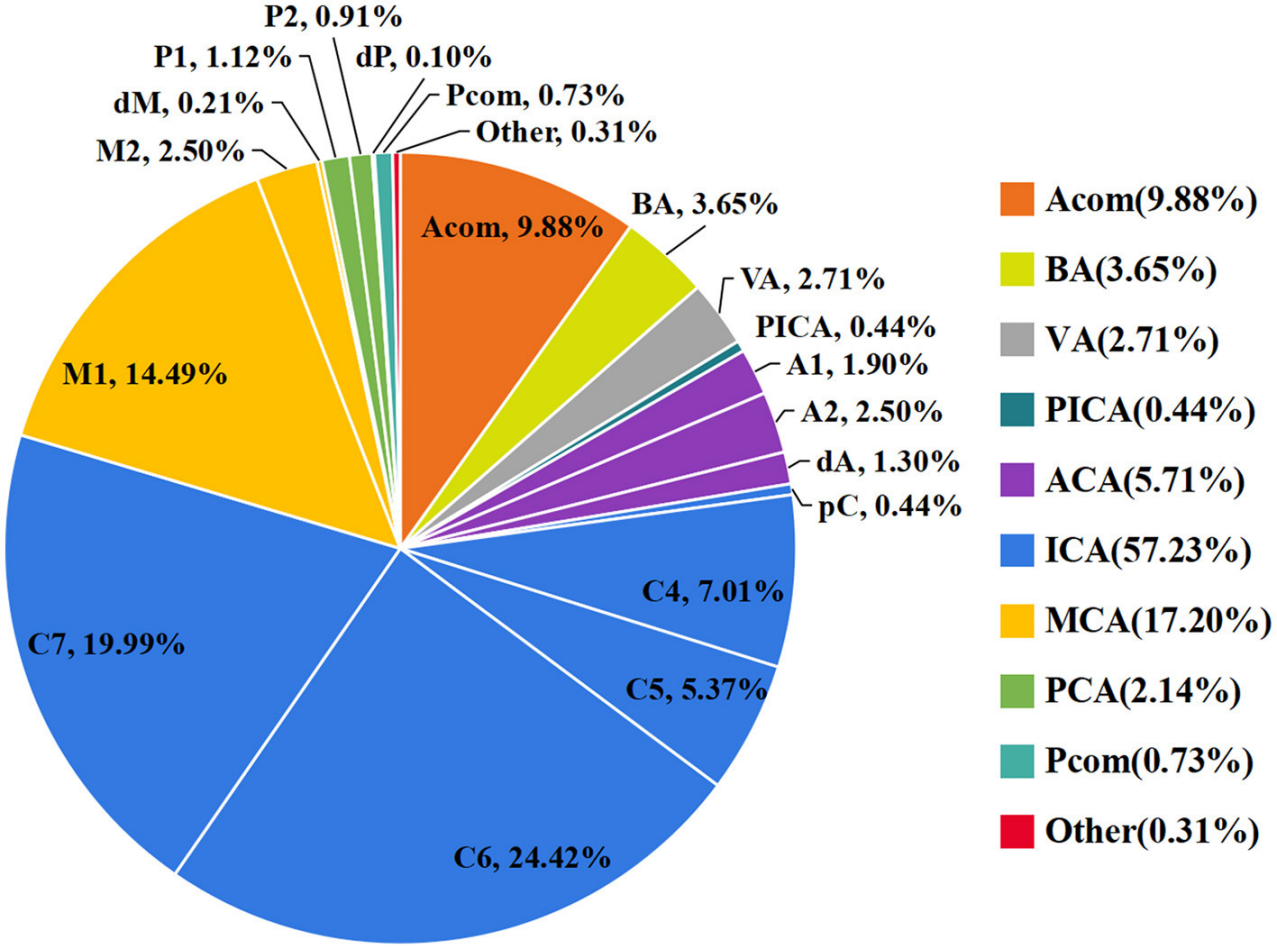
The distribution of the origins of the aneurysms.

**Figure 2:**
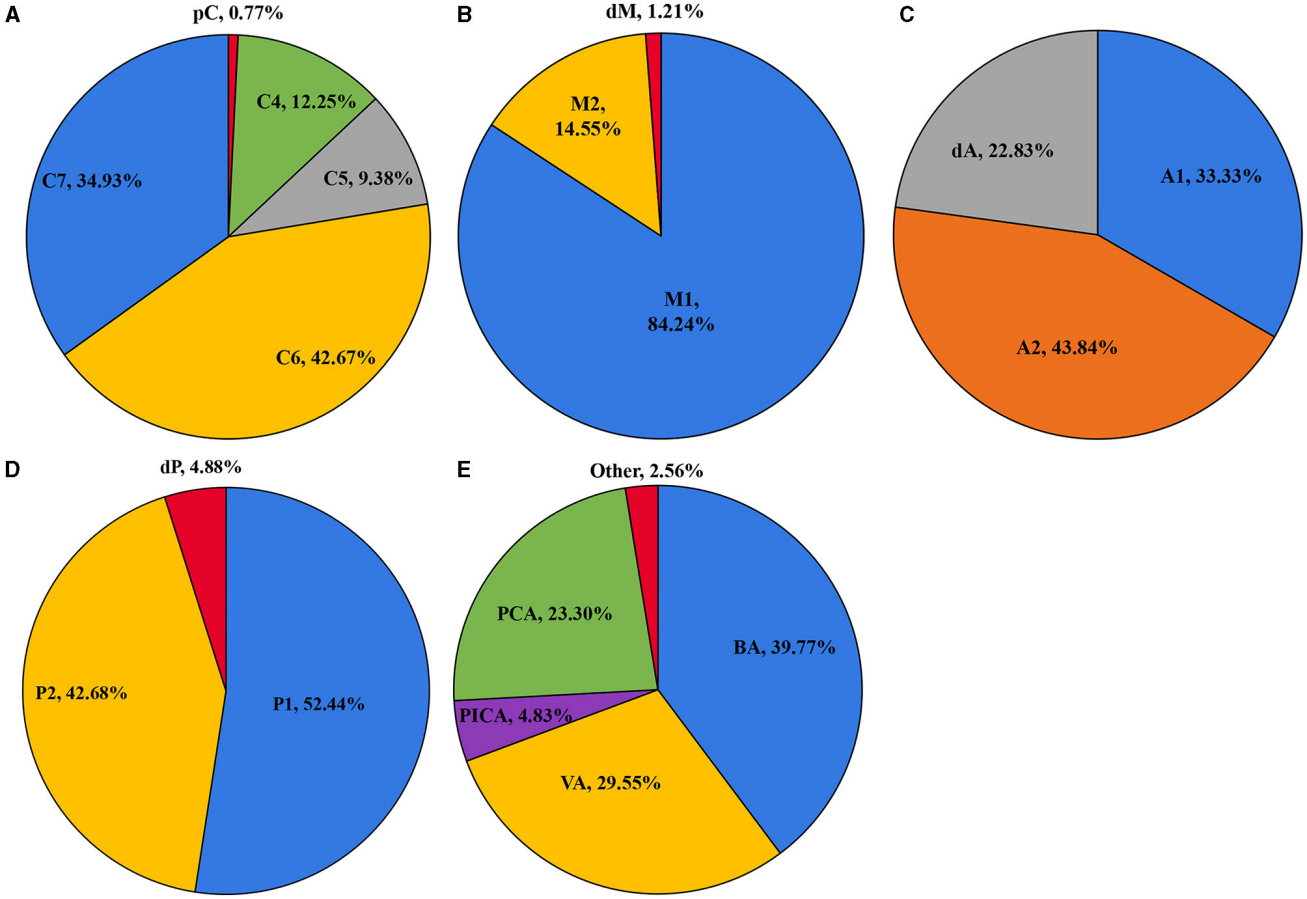
The distribution of the aneurysms of the ICA **(A)**, MCA **(B)**, ACA **(C)**, PCA **(D)**, and posterior circulation **(E)**.

As shown in Figure 1, the ICA is the most common location of IAs, with the most frequent segment being C6. Figure 2 shows the distribution of the aneurysms of each artery segment. Table 3 shows the distribution of the ruptured and unruptured IAs.

### Ruptured aneurysm proportions of different origins

**Figure 3:**
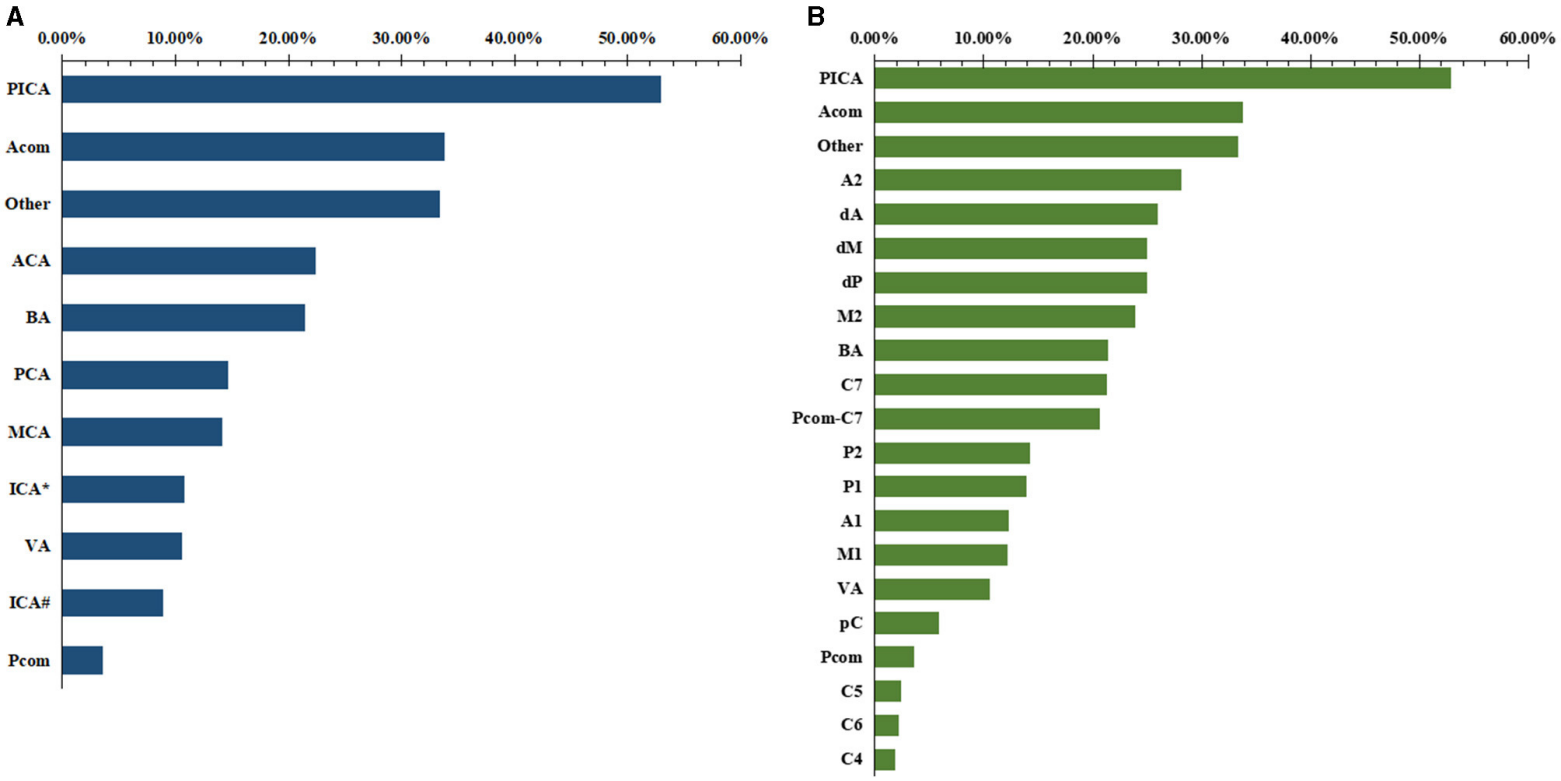
The ranking of the proportions of ruptured aneurysms of different locations. **(A)** Cerebral artery. **(B)** arterial segment.

We analyzed the ruptured aneurysm proportions of different cerebral arteries and different arterial segments (Table 3). The ranking of the ruptured aneurysm proportions is shown in Figure 3.

The ruptured aneurysm proportion of C7 (21.25%) was significantly higher than that of C4 (5.88%, *p* < 0.001), C5 (1.86%, *p* < 0.001), and C6 (2.24%, *p* < 0.001). The ruptured aneurysm proportion of M2 (23.96%) was significantly higher than that of M1 (12.23%, *p* = 0.002). The ruptured aneurysm proportion of A2 (28.13%) was significantly higher than that of A1 (12.33%, *p* = 0.013).

### Rupture risk factors analysis of IAs

Factors such as bifurcation, presence of a daughter sac, size, age, presence of multiple aneurysms, gender, parent artery stenosis, artery of origin, hyperlipidemia, diabetes mellitus, hypertension, and smoking history were included in the analysis.

A comparative analysis between the ruptured and unruptured IAs was conducted. As shown in Table 2, gender (*p* = 0.066), age (*p* = 0.974), diabetes (*p* = 0.601), hypertension (*p* = 0.359), and smoking history (*p* = 0.312) were not significantly associated with rupture. Non-hyperlipidemia (*p* < 0.001), bifurcation (*p* < 0.001), larger size (*p* = 0.001), single aneurysm (*p* < 0.001), presence of daughter aneurysms (*p* < 0.001) and non-stenotic proximal parent artery (*p* = 0.011) were found to be risk factors associated with rupture through a logistic regression analysis (Table 2).

The segments of the origins of IAs can be a risk factor according to the logistic regression analysis. For ICA IAs, C7 was an independent risk factor for IA rupture compared with C6 (*p* < 0.001, OR: 7.569; 95% CI: 4.540–12.618). For MCA IAs, dM1 was correlated with a higher rupture risk than M1 IAs (*p* = 0.044, OR: 1.961; 95% CI: 1.020–3.770). For ACA IAs, dA1 IAs were correlated with a higher rupture risk than A1 (*p* = 0.014, OR: 3.182; 95% CI: 1.264–8.013). For PCA IAs, different segment positions were not considered independent risk factors (*p* = 0.855). Other independent risk factors of the ICA, MCA, ACA, and PCA are shown in Supplementary Tables 1–4.

### Risk analysis based on PSM

Non-hyperlipidemia, bifurcation, diameter, single aneurysm, presence of daughter aneurysms, and non-stenotic proximal parent artery were the risk factors included in the PSM. A total of 776 aneurysms were selected from all aneurysms. The segments of the origins of IAs were, after further analysis, found to be a risk factor for rupture. The proportions of C4 (*p* = 0.002), C5 (*p* = 0.015), C6 (*p* < 0.001), and M1 (*p* < 0.001) in the unruptured group were significantly higher than those in the ruptured group. The proportions of C7 (*p* < 0.001), the ACoA (*p* = 0.002), and the PICA (*p* = 0.011) in the ruptured group were significantly higher than those in the unruptured group. Although the ACA (*p* = 0.028) had a higher proportion in the ruptured group, A1 (*p* = 0.203), A2 (*p* = 0.109), and dA (*p* = 0.387) had no significant difference in the two groups. More details are provided in Table 4.

**Table 4:** The distribution of aneurysms of the two groups after propensity score matching.

**Location**	**UAs/all UAs,% (*n*)**	**RAs/all RAs,%(*n*)**	***p*-value**
Total	100.00% (388)	100.00% (388)	–
ACoA	24.42% (54)	13.92% (87)	0.002
BA	4.12% (16)	5.15% (20)	0.495
VA	3.61% (14)	2.84% (11)	0.325
PICA	0.26% (1)	2.32% (9)	0.011
ACA	6.19% (24)	10.57% (41)	0.028
A1	0.77% (3)	1.80% (7)	0.203
A2	3.09% (12)	5.41% (21)	0.109
dA	2.32% (9)	3.35% (13)	0.387
ICA^#^	44.33% (172)	37.37% (145)	0.049
pC	0.26% (1)	0.26% (1)	0.547
C4	5.15% (20)	1.29% (5)	0.002
C5	4.12% (16)	1.29% (5)	0.015
ICA^*^	34.79% (135)	34.54% (134)	0.940
C6	18.30% (71)	4.64% (18)	<0.001
C7	16.49% (64)	29.90% (116)	<0.001
MCA	25.77% (100)	15.72% (61)	0.001
M1	22.16% (86)	11.86% (46)	<0.001
M2	3.61% (14)	3.35% (13)	0.845
dM	0.00% (0)	0.52% (2)	0.499
PCA	0.77% (3)	2.32% (9)	0.146
P1	0.26% (1)	1.29% (5)	0.219
P2	0.52% (2)	0.77% (3)	1.000
dP	0.00% (0)	0.26% (1)	1.000
PcoA	0.77% (3)	0.26% (1)	0.616
Other	0.26% (1)	1.03% (4)	0.373

RAs, ruptured intracranial aneurysms; UAs, unruptured intracranial aneurysms; ICA, internal carotid artery; ACA, anterior cerebral artery; ACoA, anterior communicating artery; MCA, middle cerebral artery; PCA, posterior cerebral artery; PcoA, posterior communicating artery; BA, basilar artery; VA, vertebral artery; PICA, posterior inferior cerebellar artery. pC, C1 to C3; dM, M3 to M5; dA, A3 to A5; Other, superior cerebellar artery, anterior inferior cerebellar artery, and ophthalmic artery.

IQR, interquartile range.

^#^All aneurysms in the ICA.

^*^All intracranial aneurysms in the ICA.

## Discussion

The origins of IAs and their imaging characteristics are well-known factors associated with rupture. However, to date, studies have focused only on the artery of origin and not the specific arterial segment. In addition, the contribution of preaneurysmal arterial stenosis has rarely been described. Our large-scale study showed that a segment-specific evaluation of aneurysm origin may play an important role in aneurysm instability, with C7, M2, dM, A2, and dA IAs having a higher rupture risk. These results provide new information about the assessment of IA stability, which was not reported previously.

Greving et al. (6) analyzed six prospective cohort studies and found that the ICA and MCA were the most common locations of IAs but had a low rupture risk compared to the ACoA, ACA, BA, and PCA. However, our results showed that several specific segments of the ICA and MCA (C7, M2, and dM) had a very high rupture risk, only second to the ACoA.

The reason behind C7 IAs having a high rupture risk may be due to a complex hemodynamic environment caused by higher blood flow velocities and smaller lumen areas (13). The high rupture risk of C7 IAs may be masked by the low risk of C6 aneurysms. Detmer et al. (14) showed a high risk of rupture of carotid bifurcation aneurysms, which was consistent with our results. A Japanese cohort study (15) had findings similar to ours, showing that C7-Pcom origin IAs were at a higher risk of rupture, whereas other ICA IAs were at a lower risk of rupture.

Although the size of the M2 and M1 IAs were similar in the current study, the smaller diameter (16) and thinner wall (17, 18) of M2 arterial segments likely contributed to increased M2 IA instability. Our study also found that the IAs at dA1 had a much higher risk than the A1 IAs. Previous studies of ACA origin IAs (19, 20) had shown that A1, A2, or distal ACA (A3 or beyond) aneurysms had thin walls, complex hemodynamics, and were more likely to rupture at smaller sizes than aneurysms at other locations. Lehecka et al. (21) speculated that A2 IAs had a higher rupture risk because of the perforator origin, broad base configuration, thin walls, and abnormal hemodynamics. In addition, dA1 IAs had a smaller parent artery diameter compared to A1 IAs (22), which may be associated with increased IA instability (19, 23, 24).

Similar to prior studies, we found a high rupture risk of the posterior circulation origin IAs (1, 6); however, we also found that only the BA and PICA showed a high risk of rupture compared to the mean risk of all IAs. Similar to other studies (25, 26), BA IAs were the most common posterior circulation aneurysms, accounting for 39.8% in our study. The high prevalence of BA IAs may have contributed to an overestimation of the overall posterior circulation IA rupture risk. In addition, considering the small data on posterior circulation aneurysms, further verification may be necessary.

Similar to previous studies, the risk factors for IA rupture included bifurcation origin, presence of a daughter sac, large diameter, increased age, and no stenosis of the parent artery (1, 6, 9, 14, 25, 27, 28). The aneurysms with a non-stenotic parent artery usually exhibited lower wall shear stress (29), and this phenomenon may have led to a higher rupture risk (30) because of inflammation (31). The current study did not find female sex to be a risk factor for rupture. Age and menopause have a significant impact on aneurysms in female patients, which may explain the discrepancies in the results of different studies regarding sex as a risk factor for rupture.

After adjusting for the risks attributed to aneurysm rupture, C7 IAs, ACoA IAs, and PICA IAs showed a high risk of rupture and C4 IAs, C5 IAs, C6 IAs, and M1 IAs showed a low risk of rupture. This finding indicates that the origin of an aneurysm is also a risk factor. However, after PSM, the number of aneurysms included in the study was significantly reduced, necessitating further investigation of the results.

## Limitations

There were a number of limitations of this study. First, this was a single-center retrospective study including a Chinese cohort. Most of the patients included were local residents, especially those with ruptured aneurysms. In addition, many unruptured aneurysms are found through physical examination or in relation to other reasons. These aspects led to population bias in our data, which may not translate to other populations. Second, most aneurysms were treated soon after discovery, with patients not undergoing a long-term follow-up, which may have resulted in bias toward those aneurysms deemed to not require initial treatment. Third, every aneurysm was represented in this study as a single data point. All risk factors were summaries of existing cases, and there was a lack of follow-up observation and strict control of variables. The study could not completely exclude the interference of confounding factors. Aneurysms were so numerous and rare in some segments that they had to be merged into others and could not be assessed for risk. In addition, patients with prior subarachnoid hemorrhage were not included in the study because many patients were diagnosed at other hospitals and did not have complete images and medical records of ruptured aneurysms. This study could not determine the effect of previous subarachnoid hemorrhage on aneurysm rupture, potentially contributing to bias. Therefore, further investigation in a large prospective longitudinal cohort is necessary.

## Conclusion

Beyond the already established risk associated with IA rupture at the artery of origin, this study showed that further risk can be attributed to specific segments of origin, which might improve predictions of aneurysm instability. Aneurysms originating from the ACoA, BA, and PICA and A2, dA, C7, and M2 indicated a higher rupture risk. Aneurysms originating from C4, C5, and C6 indicated a lower rupture risk. C7 IAs, ACoA IAs, and PICA IAs appeared to be independent risk factors. A multicenter prospective cohort study can better refine the findings of this study.

## Data Availability

The corresponding author will provide data supporting the conclusions of this article upon reasonable request.
