# Supplementary material for: Risk analysis of intracranial aneurysm rupture based on the arterial segment of origin
Source: Front Neurol. 2024 Aug 21;15:1339144. doi: 10.3389/fneur.2024.1339144 (PMC11371744; doi:10.3389/fneur.2024.1339144)
Supplement: Supplementary file 1 [file Table_1.DOCX]

|  | Rupture,%  (n=93) | Unrupture,%  (n=567) | P | p* | OR  (95%CI) |
| --- | --- | --- | --- | --- | --- |
| Gender |  |  |  |  |  |
| male | 44.09% | 41.09% | 0.587 | - | - |
| female | 55.91% | 58.91% |  |  |  |
| No of aneurysms |  |  |  |  |  |
| single | 79.57% | 53.97% | <0.001 | <0.001 | 3.585  (1.878-6.846) |
| multiple# | 20.43% | 46.03% |  |  |  |
| Daughter aneurysms |  |  |  |  |  |
| without# | 61.29% | 97.70% | <0.001 | <0.001 | 37.319  (15.899-87.597) |
| with | 38.71% | 2.30% |  |  |  |
| Situation |  |  |  |  |  |
| non-bifurcation# | 10.75% | 23.99% | 0.004 | 0.004 | 3.411  (1.488-7.817) |
| bifurcation | 89.25% | 76.01% |  |  |  |
| Size* | 5.35  (4.12,7.40) | 3.90  (2.85,5.90) | <0.001 | 0.712 | 1.015  (0.937-1.100) |
| Age * | 56 (47,63) | 59 (52,64) | 0.008 | 0.017 | 0.967  (0.940-0.994) |
| Parent artery |  |  |  |  |  |
| non-stenotic | 95.70% | 85.36% | 0.010 | 0.047 | 3.154  (1.013-9.817) |
| stenotic# | 4.360% | 14.64% |  |  |  |
| Blood lipids |  |  |  |  |  |
| hyperlipidemia# | 5.38% | 18.87% | 0.001 | 0.002 | 5.569  (1.876-16.536) |
| non-hyperlipidemia | 94.62% | 81.13% |  |  |  |
| Blood glucose |  |  |  |  |  |
| diabetes | 15.05% | 15.52% | 0.908 | - | - |
| non-diabetes | 84.95% | 84.48% |  |  |  |
| Blood pressure |  |  |  |  |  |
| hypertension | 67.74% | 71.08% | 0.513 | - | - |
| non-hypertension | 32.26% | 28.92% |  |  |  |
| Smoking history |  |  |  |  |  |
| smoking | 21.51% | 25.04% | 0.462 | - | - |
| non-smoking | 78.49% | 74.96% |  |  |  |
| Location |  |  |  |  |  |
| M1# | 73.12% | 86.07% | 0.001 | 0.044 | 1.961  (1.020-3.770) |
| dM1 | 26.88% | 13.93% |  |  |  |

**Supplemental Table 1** The result of the logistic regression analysis about the distribution of risk factors and IAs location in MCA between two groups

dM1: the segment at the distal of M1

p：chi-square test or Mann-Whitney U test; p*：multivariate logistic regression

OR: odds ratio

|  | Rupture,%  (n=184) | Unrupture,%  (n=1520) | p | p* | OR  (95%CI) |
| --- | --- | --- | --- | --- | --- |
| Gender |  |  |  |  |  |
| male | 26.63% | 31.25% | 0.200 | - | - |
| female | 73.37% | 68.75% |  |  |  |
| No of aneurysms |  |  |  |  |  |
| single | 73.37% | 58.95% | <0.001 | <0.001 | 3.101  (2.014-4.776) |
| multiple# | 26.63% | 41.05% |  |  |  |
| Daughter aneurysms |  |  |  |  |  |
| without# | 69.02% | 99.01% | <0.001 | <0.001 | 23.364  (11.874-45.971) |
| with | 30.98% | 0.99% |  |  |  |
| Situation |  |  |  |  |  |
| non-bifurcation# | 61.33% | 89.54% | <0.001 | 0.001 | 2.053  (1.353-3.116) |
| bifurcation | 39.67% | 10.46% |  |  |  |
| Size* | 3.15  (2.45,4.50) | 5.05  (3.79,7.2775) | <0.001 | 0.006 | 1.065  (1.019-1.114) |
| Age * | 62 (51.25,69) | 57 (50,63) | <0.001 | 0.014 | 1.023  (1.005-1.041) |
| Parent artery |  |  |  |  |  |
| non-stenotic | 75.54% | 76.32% | 0.816 | - | - |
| stenotic | 24.46% | 23.68% |  |  |  |
| Blood lipids |  |  |  |  |  |
| hyperlipidemia# | 8.70% | 20.59% | <0.001 | <0.001 | 3.426  (1.845-6.360) |
| non-hyperlipidemia | 91.30% | 79.41% |  |  |  |
| Blood glucose |  |  |  |  |  |
| diabetes | 18.48% | 16.58% | 0.515 | - | - |
| non-diabetes | 81.52% | 83.42% |  |  |  |
| Blood pressure |  |  |  |  |  |
| hypertension | 60.33% | 62.04% | 0.651 | - | - |
| non-hypertension | 39.67% | 37.96% |  |  |  |
| Smoking history |  |  |  |  |  |
| smoking | 18.48% | 20.39% | 0.541 | - | - |
| non-smoking | 81.52% | 79.61% |  |  |  |
| Location |  |  |  |  |  |
| C6# | 11.11% | 53.22% | <0.001 | <0.001 | 7.569  (4.540-12.618) |
| C7 | 86.24% | 35.10% |  |  |  |

**Supplemental Table 2** The result of the logistic regression analysis about the distribution of risk factors and IAs location in ICA between two groups

p：chi-square test or Mann-Whitney U test; p*：multivariate logistic regression

OR: odds ratio

|  | Rupture,%  (n=49) | Unrupture,%  (n=170) | P | p* | OR  (95%CI) |
| --- | --- | --- | --- | --- | --- |
| Gender |  |  |  |  |  |
| male | 42.86% | 38.24% | 0.559 | - | - |
| female | 57.14% | 61.76% |  |  |  |
| No of aneurysms |  |  |  |  |  |
| single | 81.63% | 56.47% | 0.001 | 0.001 | 4.587  (1.819-11.567) |
| Multiple# | 18.37% | 43.53% |  |  |  |
| Daughter aneurysms |  |  |  |  |  |
| Without# | 79.59% | 97.06% | <0.001 | 0.003 | 8.633  (2.115-35.243) |
| with | 20.41% | 2.94% |  |  |  |
| Situation |  |  |  |  |  |
| non-bifurcation | 82.76% | 74.11% | 0.001 | 0.020 | 2.449  (1.153-5.199) |
| Bifurcation# | 17.24% | 25.89% |  |  |  |
| Size* | 4.25  (3.22,6.24) | 3.04  （2.1875，4.1225） | <0.001 | 0.040 | 1.161  (1.007-1.339) |
| Age * | 61(53.5,65.5) | 58 (50,64) | 0.174 | - | - |
| Parent artery |  |  |  |  |  |
| non-stenotic | 95.91% | 90.00% | 0.313 | - | - |
| stenotic | 4.09% | 10.00% |  |  |  |
| Blood lipids |  |  |  |  |  |
| hyperlipidemia | 10.20% | 12.35% | 0.682 | - | - |
| non-hyperlipidemia | 89.80% | 87.65% |  |  |  |
| Blood glucose |  |  |  |  |  |
| diabetes | 14.29% | 15.29% | 0.862 | - | - |
| non-diabetes | 85.71% | 84.71% |  |  |  |
| Blood pressure |  |  |  |  |  |
| hypertension | 67.35% | 70.59% | 0.663 | - | - |
| non-hypertension | 32.65% | 29.41% |  |  |  |
| Smoking history |  |  |  |  |  |
| smoking | 22.45% | 24.71% | 0.745 | - | - |
| non-smoking | 77.55% | 75.29% |  |  |  |
| Location |  |  |  |  |  |
| A1# | 18.37% | 37.65% | 0.012 | 0.015 | 3.079  (1.243-7.625) |
| dA1 | 81.63% | 62.35% |  |  |  |

**Supplemental Table 3** The result of the logistic regression analysis about the distribution of risk factors and IAs location in ACA between two groups

dA1: the segment at the distal of A1

p：chi-square test; p*：multivariate logistic regression

OR: odds ratio

|  | Rupture,%  (n=49) | Unrupture,%  (n=170) | p | p* | OR  (95%CI) |
| --- | --- | --- | --- | --- | --- |
| Gender |  |  |  |  |  |
| male | 41.67% | 37.14% | 0.765 | - | - |
| female | 58.33% | 62.86% |  |  |  |
| Number of aneurysms |  |  |  |  |  |
| single | 66.67% | 42.86% | 0.224 | - | - |
| multiple | 33.33% | 57.14% |  |  |  |
| Daughter aneurysms |  |  |  |  |  |
| without | 75.00% | 92.86% | 0.162 | - | - |
| with | 25.00% | 7.14% |  |  |  |
| Situation |  |  |  |  |  |
| non-bifurcation | 91.67% | 84.29% | 0.821 | - | - |
| bifurcation | 8.33% | 15.71% |  |  |  |
| Size* | 4.525  (3.44,9.565) | 2.955  (1.8925,4.0225) | 0.003 | 0.020 | 1.201  （1.029-1.402） |
| Age * | 59 (47,70.25) | 60 (51,65) | 0.901 | - | - |
| Parent artery |  |  |  |  |  |
| non-stenotic | 91.67% | 60.00% | 0.073 | - | - |
| stenotic | 8.33% | 40.00% |  |  |  |
| Blood lipids |  |  |  |  |  |
| hyperlipidemia | 0.00% | 18.57% | 0.198 | - | - |
| non-hyperlipidemia | 100.00% | 81.43% |  |  |  |
| Blood glucose |  |  |  |  |  |
| diabetes | 0.00% | 14.29% | 0.344 | - | - |
| non-diabetes | 100.00% | 85.71% |  |  |  |
| Blood pressure |  |  |  |  |  |
| hypertension | 83.33% | 60.00% | 0.220 | - | - |
| non-hypertension | 16.67% | 40.00% |  |  |  |
| Smoking history |  |  |  |  |  |
| smoking | 8.33% | 30.00% | 0.225 | - | - |
| non-smoking | 91.67% | 70.00% |  |  |  |
| Location |  |  |  |  |  |
| P1 | 50.00% | 52.86% | 0.855 | - | - |
| dP1 | 50.00% | 47.14% |  |  |  |

**Supplemental Table 4** The result of the logistic regression analysis about the distribution of risk factors and IAs location in PCA between two groups

dP1: the segment at the distal of P1

p：chi-square test or Mann-Whitney U test; p*：multivariate logistic regression

OR: odds ratio
